# MiRNA-124-3p.1 sensitizes hepatocellular carcinoma cells to sorafenib by regulating FOXO3a by targeting AKT2 and SIRT1

**DOI:** 10.1038/s41419-021-04491-0

**Published:** 2022-01-10

**Authors:** Zhe-bin Dong, Heng-miao Wu, Yi-cheng He, Zhong-ting Huang, Yi-hui Weng, Hong Li, Chao Liang, Wei-ming Yu, Wei Chen

**Affiliations:** 1 Department of General Surgery, the Affiliated Lihuili Hospital, Ningbo University, Ningbo, 315000 PR China; 2 Cancer Institute of Integrated Traditional Chinese and Western Medicine, Zhejiang Academy of Traditional Chinese Medicine, Tongde Hospital of Zhejiang Province, Hangzhou, 310012 PR China

**Keywords:** Cancer therapeutic resistance, Tumour-suppressor proteins, Apoptosis, miRNAs, Predictive markers

## Abstract

As a multikinase inhibitor, sorafenib is commonly used to treat patients with advanced hepatocellular carcinoma (HCC), however, acquired resistance to sorafenib is a major obstacle to the effectiveness of this treatment. Thus, in this study, we investigated the mechanisms underlying sorafenib resistance as well as approaches devised to increase the sensitivity of HCC to sorafenib. We demonstrated that miR-124-3p.1 downregulation is associated with early recurrence in HCC patients who underwent curative surgery and sorafenib resistance in HCC cell lines. Regarding the mechanism of this phenomenon, we identified FOXO3a, an important cellular stress transcriptional factor, as the key factor in the function of miR-124-3p.1 in HCC. We showed that miR-124-3p.1 binds directly to *AKT2* and *SIRT1* to reduce the levels of these proteins. Furthermore, we showed that AKT2 and SIRT1 phosphorylate and deacetylate FOXO3a. We also found that miR-124-3p.1 maintains the dephosphorylation and acetylation of FOXO3a, leading to the nuclear location of FOXO3a and enhanced sorafenib-induced apoptosis. Moreover, the combination of miR-124-3p.1 mimics and sorafenib significantly enhanced the curative efficacy of sorafenib in a nude mouse HCC xenograft model. Collectively, our data reveal that miR-124-3p.1 represents a predictive indicator of early recurrence and sorafenib sensitivity in HCC. Furthermore, we demonstrate that miR-124-3p.1 enhances the curative efficacy of sorafenib through dual effects on FOXO3a. Thus, the miR-124-3p.1-FOXO3a axis is implicated as a potential target for the diagnosis and treatment of HCC.

## Introduction

Hepatocellular carcinoma (HCC) ranks fifth in terms of the global cases of cancer and is the second leading cause of cancer-related death in men worldwide [1]. However, many patients present with advanced HCC, when surgery or regional treatments are invalid, and systemic therapy is required. Unlike other tumors, HCC rarely responds to chemotherapy, thus, targeted therapy is likely to be a better option. Sorafenib is a multikinase inhibitor that suppresses tumor cell proliferation and angiogenesis and promotes tumor cell apoptosis. Sorafenib is also the most commonly used first-line target therapy for advanced HCC [2]. In two large clinical trials, sorafenib was shown to extend survival by 2–3 months for HCC patients in both Western and Eastern populations [3, 4]. Despite its efficacy, drug resistance emerges soon after initial treatment, although the underlying mechanisms is still unclear. Consequently, there is an urgent need to find new therapeutic targets. Recent studies have indicated that microRNAs (miRNAs) play an important role in the development of sorafenib resistance [5, 6].

MiRNAs are a series of short (12–24 nucleotides) non-coding single-stranded RNA molecules encoded by endogenous genes. MiRNAs regulate gene expression mainly by binding to the non-coding region at the 3′-end of the mRNA resulting in degradation of the target RNA termination of its translation [7]. Accumulating evidence indicates that the cancer progression and the development of drug resistance are closely related to differential expression of miRNAs [8, 9]. MiR-124-3p.1 was identified as a potential tumor suppressor associated with diverse processes including proliferation, apoptosis, and metastasis [10–12]. Recent studies have revealed that low miR-124-3p.1 expression levels are associated with poor survival among patients with HCC [13]. In addition, it has been confirmed that miR-124-3p.1 sensitizes CD133^+^ cells to cisplatin-induced cytotoxicity in HCC [14]. However, the potential involvement of miR-124-3p.1 in sorafenib resistance remains to be established. Understanding the mechanisms of by which miR-124-3p.1 is involved in the development of drug resistance may facilitate the development of novel approaches to HCC therapy. We here investigated the relationship between miR-124-3p.1 and sorafenib resistance in HCC.

The protein kinase B family divided into three subtypes, AKT1, AKT2, AKT3. Activation of AKT has been shown to be related to apoptosis in cancers [15]. Moreover, expression of AKT2, but not AKT1 or AKT3, was found to be related to the prognosis of HCC patients [16]. Furthermore, AKT2 overexpression was associated with the resistance to erlotinib in pancreatic cancer cell lines [17], indicating that AKT2 is involved in the occurrence of drug resistance. Garten et al. found that sorafenib-induced apoptosis in HCC was reversed by overexpression of Sirtuin 1 (SIRT1) [18], which is a nicotinamide adenine dinucleotide-dependent enzyme that is frequently overexpressed in HCC, and promotes tumor occurrence, metastasis, and chemoresistance [19]. It is worth noting that both AKT2 and SIRT1 are related to tumor prognosis and drug resistance, and also gene targets of miR-124-3p.1. Therefore, we speculated that AKT2 and SIRT1 are the main downstream targets of miR-124-3p. 1 and play an important role in the mechanisms underlying its antitumor effects and ability to reverse drug resistance.

FOXO3a, which is a member of the FOXO subfamily of forkhead transcription factors (TFs), mediates a variety of cellular processes including apoptosis, cell cycle progression, DNA damage, and tumorigenesis and is suggested to play a pivotal role as a tumor suppressor in HCC [20, 21]. FOXO3a is known to bind and activate the promoter of the pro-apoptotic gene Bim to induce apoptosis [22]. It has also been reported that Bim is involved in sorafenib-induced apoptosis [23]. Since AKT2 and SIRT1 are recognized as important upstream regulators of FOXO3a [24], we hypothesized that FOXO3a is also involved in the antitumor effect of miR-124-3p.1 and sorafenib-induced apoptosis.

In the current study, our results indicate that miRNA-124-3p.1 sensitizes HCC cells to sorafenib-induced apoptosis through regulation of the phosphorylation and deacetylation of FOXO3a by targeting AKT2 and SIRT1. These findings may provide a new predictor of sorafenib efficacy and potential therapeutic targets in HCC.

## Results

### MiR-124-3p.1 is downregulated in human HCC tissues and cell lines and low expression predicts early recurrence of HCC

**Fig. 1:**
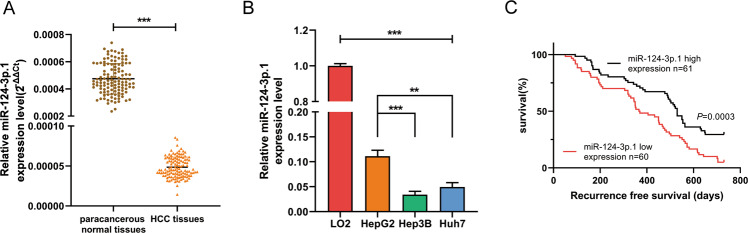
MiR-124-3p.1 is downregulated in human HCC tissues and cell lines and low expression predicts early recurrence of HCC. **A** qRT-PCR analysis of miR-124-3p.1 expression in human HCC tissues and corresponding paracancerous normal tissues (*n* = 121, *t*-test). **B** qRT-PCR analysis of miR-124-3p.1 expression in normal cells (LO2) and HCC cells (HepG2, Hep3B and Huh-7, *t*-test). **C** The relationship between expression of miR-124-3p.1 and recurrence-free survival of HCC patients in the Affiliated Lihuili Hospital, Ningbo University. Data represent mean ± SD (*n* = ≥3 experiments). *P < 0.05, **P < 0.01, ***P < 0.001.

We first analyzed the expression of miR-124-3p.1 in 121 pairs of tumor and paracancerous normal tissue samples from HCC patients and several HCC cell lines by qRT-PCR. The results revealed downregulated expression of miR-124-3p.1 in the tumor group compared with the paracancerous normal tissues (Fig. 1A). In addition, we showed differences in the expression levels of miR-124-3p.1 between six HCC cell lines and found that HepG2 cells expressed higher levels of miR-124-3p.1 than the other cell lines. Three representative cell lines (HepG2, Hep3B and Huh-7) were selected for use in the subsequent experiments (Supplementary Fig 1). However, miR-124-3p.1 expression in HCC cell lines was significantly lower than that of normal hepatic cells (Fig. 1B). We further analyzed the relationship between miR-124-3p.1 and HCC recurrence. HCC patients (*n* = 121) were separated into two groups by the median miR-124-3p.1 expression level. As shown in Fig. 1C, patients with higher miR-124-3p.1 expression (*n* = 61, including the median) showed longer recurrence-free survival than patients with lower miR-124-3p.1 expression (*n* = 60).

Further analysis of the correlation between miR-124-3p.1 expression and clinicopathological parameters showed significant differences in HBV infection, microvascular invasion (MVI), and alpha-fetoprotein (AFP) levels between the high and low miR-124-3p.1 expression groups (Supplementary Table 1). In univariate analyses, expression levels of miR-124-3p.1 and AFP and MVI were identified as significant prognostic factors for recurrence-free survival (Supplementary Table 2). However, in multivariate analyses, only miR-124-3p.1 expression level was identified as a prognostic factor for recurrence-free survival (Supplementary Table 3). These results indicated that miR-124-3p.1 represents a biomarker of early HCC recurrence.

### MiR-124-3p.1 enhances sorafenib-induced apoptosis

**Fig. 2:**
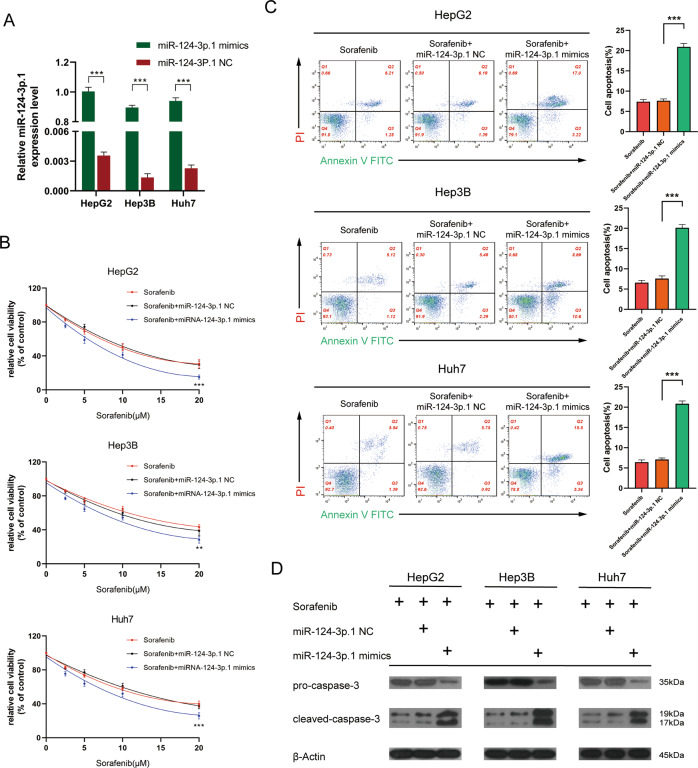
MiR-124-3p.1 enhances sorafenib-induced apoptosis. **A** qRT-PCR analysis of transfection efficiency of miR-124-3p.1 mimics in HCC cells (*t*-test). **B** Relative cell viability of HCC cells (cultured with sorafenib, sorafenib + miR-NC, sorafenib + miR-124-3p.1 mimics, two-way ANOVA). **C** Flow cytometric analysis of cell apoptosis rate in HCC cells cultured with sorafenib10 µM, sorafenib 10 µM + miR-NC, or sorafenib 10 µM + miR-124-3p.1 mimics (*t*-test). **D** Western blot analysis of pro-caspase-3 (35 kDa) and cleaved-caspased-3 (19, 17 kDa) expression in HCC cells (cultured with sorafenib10 µM, sorafenib 10 µM + miR-NC, sorafenib 10 µM + miR-124-3p.1 mimics). Data represent mean ± SD (*n* = ≥3 experiments). *P < 0.05, **P < 0.01, ***P < 0.001.

To investigate the relationship between the miR-124-3p.1 and sorafenib in HCC, we overexpressed miR-124-3p.1 in HCC cell lines by transfection with specific mimics (Fig. 2A). CCK-8 assays indicated that cells overexpressing miR-124-3p.1 were more sensitive to sorafenib compared with the control group (Fig. 2B). However, overexpression of miR-124-3p.1 alone made no effect on HCC cell viability, indicating that miR-124-3p.1 alone enhanced the sensitization of HCC cells to sorafenib without direct cytotoxic (Supplementary Fig. 2). Flow cytometric analysis showed significantly higher rates of apoptosis in the miR-124-3p.1 mimics group following sorafenib treatment compared with the rates induced in the miR-NC and untransfected control groups (Fig. 2C). Moreover, the results of the Western blotting analysis were consistent with the flow cytometry data, with higher expression of cleaved-caspase-3 in the miR-124-3p.1 mimics group compared with that than in the miR-NC group (Fig. 2D).

### MiR-124-3p.1 binds to AKT2 and SIRT1

**Fig. 3:**
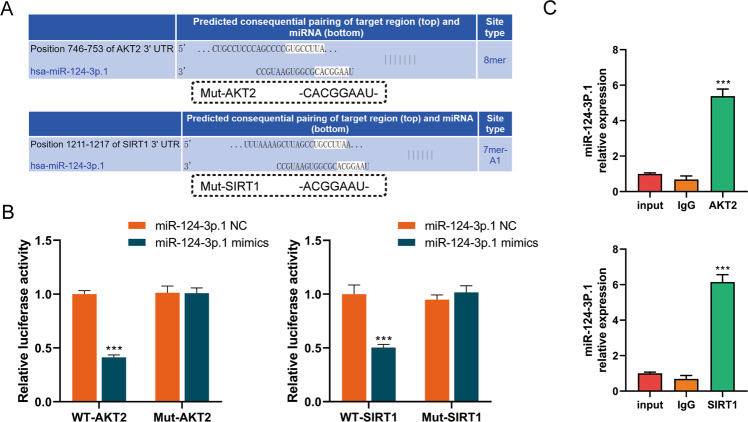
MiR-124-3p.1 binds to AKT2 and SIRT1. **A** Potential miR-124-3p.1 binding sites in the 3′-UTR of wild-type (WT) and mutant (Mut) *AKT2* and *SIRT1*. **B** Luciferase reporter assay of miR-124-3p.1 binding to SIRT1-WT/-Mut and AKT2-WT/-Mut (*t*-test). **C** RIP assays of the interaction of miR-124-3p.1 with AKT2 and SIRT1 (*t*-test). Data represent mean ± SD (*n* = ≥3 experiments). *P < 0.05, **P < 0.01, ***P < 0.001.

We used TargetScan, a public miRNA prediction database (http://www.targetscan.org/), to identify specific gene targets of miR-124-3p.1. Among the hundreds of predicted targets, we screened out AKT2 and SIRT1, which are considered to be oncogenes and related to drug resistance [17, 25–27] (Fig. 3A). Moreover, we conducted luciferase reporter assays using vectors containing the 3′-UTR segments of *AKT2* and *SITR1* including the wild-type (WT) or the mutant miR-124-3p binding sites to verify the interaction between miR-124-3p.1 and AKT2/SIRT1 in HCC. In both the AKT2 and SIRT1 groups, the luciferase activity of the WT vector group was significantly inhibited by miR-124-3p.1, whereas the luciferase activity of the mutant group was not suppressed (Fig. 3B). In addition, RNA-binding protein immunoprecipitation assay revealed higher miR-124-3p.1 expression in the AKT2 and SIRT1 groups, thus confirming that miR-124-3p.1 binds directly to AKT2 and SIRT1 in HCC cell lines (Fig. 3C). These results confirmed that *AKT2* and *SIRT1* are targets of miR-124-3p.1, which might be related to the ability of miR-124-3p.1 to promote sorafenib-mediated apoptosis.

### FOXO3a is a key factor in sorafenib-induced apoptosis

**Fig. 4:**
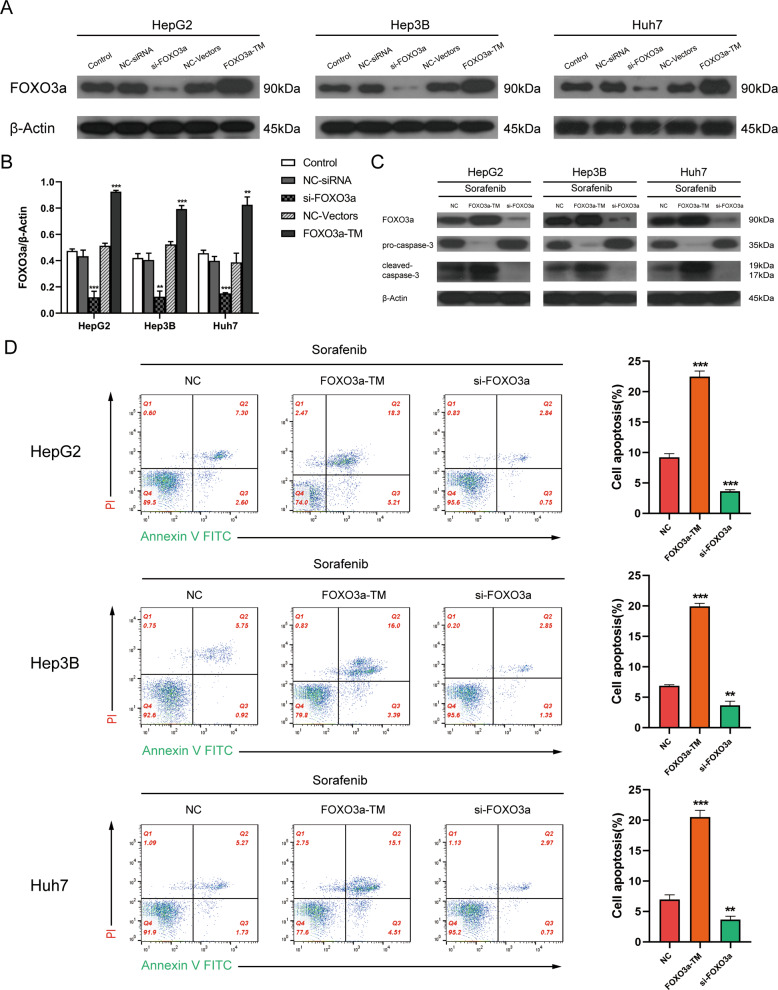
FOXO3a is a key factor in sorafenib-induced apoptosis. **A** Western blot analysis of the relative expression levels of FOXO3a (90 kDa) in HCC cells transfected with si-FOXO3a or FOXO3a-TM plasmid. **B** Densitometric analysis of FOXO3a/β-Actin ratios (*t*-test). **C** Western blot analysis of pro-caspase-3 (35 kDa) and cleaved-caspased-3 (19, 17 kDa) expression in HCC cells (cultured with sorafenib10 µM + NC, sorafenib 10 µM + FOXO3a-TM, sorafenib 10 µM + si-FOXO3a). **D** Flow cytometric analysis of cell apoptosis rate in HCC cells cultured with sorafenib10 µM + NC, sorafenib 10 µM + FOXO3a-TM, or sorafenib 10 µM + si-FOXO3a (*t*-test). Data represent mean ± SD (*n* = ≥3 experiments). *P < 0.05, **P < 0.01, ***P < 0.001.

AKT2 and SIRT1 have been reported to be common upstream regulators of FOXO3a [28, 29]. Therefore, we then explored the involvement of FOXO3a in sorafenib-induced apoptosis in HCC cell lines. We established stable FOXO3a overexpression and knockdown cell lines and detected the level of cell apoptosis by Western blotting and flow cytometry. First, the efficacy of FOXO3a-TM plasmid and si-FOXO3a was evaluated by Western blot analysis of relative FOXO3a expression levels (Fig. 4A, B). In the subsequent experiments, FOXO3a knockdown greatly decreased the expression of cleaved-caspase-3 and the rate of cell apoptosis in the sorafenib treated group, while FOXO3a overexpression had the opposite effect (Fig. 4C, D). Thus, we confirmed that FOXO3a plays a vital role in sorafenib-induced apoptosis.

### MiR-124-3p.1 promotes nuclear localization of FOXO3a by targeting AKT2

**Fig. 5:**
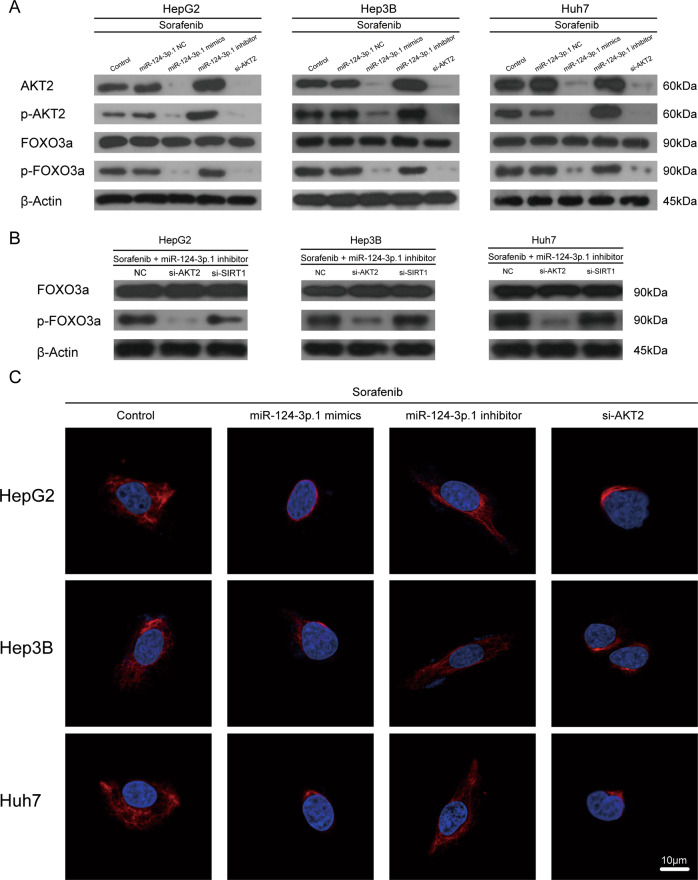
MiR-124-3p.1 promotes nuclear localization of FOXO3a by targeting AKT2. **A** Western blot analysis of AKT2 (60 kDa), phospho-AKT2 (60 kDa), FOXO3a (90 kDa), and phospho-FOXO3a (90 kDa) expression in HCC cells (cultured with sorafenib 10 µM, sorafenib 10 µM + miR-NC, sorafenib 10 µM + miR-124-3p.1 mimics, sorafenib 10 µM + miR-124-3p.1 inhibitor, or sorafenib 10 µM + si-AKT2). **B** Western blot analysis of FOXO3a (90 kDa), and phospho-FOXO3a (90 kDa) expression in HCC cells cultured with sorafenib 10 µM+miR-124-3p.1 inhibitor + NC, sorafenib 10 µM + miR-124-3p.1 inhibitor+si-AKT2, or sorafenib 10 µM+miR-124-3p.1 inhibitor+si-SIRT1. **C** Immunofluorescence staining patterns of AF555-labeled FOXO3a showing the subcellular localization of FOXO3a in HCC cells (cultured with sorafenib 10 µM, sorafenib 10 µM + miR-124-3p.1 mimics, sorafenib 10 µM + miR-124-3p.1 inhibitor, or sorafenib 10 µM + si-AKT2). Each experiment was performed in triplicate.

FOXO3a has been shown to function as a downstream target of AKT2 and to phosphorylated by AKT2, resulting in cytoplasmic localization of FOXO3a [30]. In our study, Western blot analysis revealed that p-FOXO3a expression decreased significantly in three HCC cell lines following siRNA-induced silencing of AKT2 compared with the control group, thus confirming that AKT2 is the most important upstream regulator of FOXO3a phosphorylation (Fig. 5A). Further studies revealed a significant decrease in AKT2 and AKT2 phosphorylation following overexpression of miR-124-3p.1. Moreover, phosphorylation of FOXO3a, the downstream target of AKT2, was also downregulated following miR-124-3p.1 overexpression (Fig. 5A). Following transfection of HCC cells with the miR-124-3p.1 inhibitor, the protein levels of AKT2, p-AKT2, and p-FOXO3a were increased remarkably compared to the levels detected in the control group (Fig. 5A). We further demonstrated that miR-124-3p.1 induced FOXO3a dephosphorylation by targeting AKT2. After treatment of three HCC cells with Sorafenib + miR-124-3p.1 inhibitor, p-FOXO3a expression was significantly higher in the si-SIRT1 group than that in the si-AKT2 group (Fig. 5B). These results indicated that miR-124-3p.1 decreases the FOXO3a phosphorylation by suppressing the expression of AKT2 and p-AKT2.

By using immunofluorescence staining, we detected the subcellular localization of FOXO3a in HCC cells treated with miR-124-3p.1 mimics, miR-124-3p.1 inhibitor and si-AKT2. Laser scanning confocal microscope scanning of AF555-labeled FOXO3a revealed that si-AKT2 and miR-124-3p.1 mimics promoted nuclear accumulation of FOXO3a, whereas inhibition of miR-124-3p.1 resulted in cytoplasmic localization of FOXO3a (Fig. 5C). Based on these findings, we concluded that the expression of phosphorylated AKT2 and FOXO3a in HCC cells was decreased by miR-124-3p.1, and induced nuclear translocalization of FOXO3a.

### MiR-124-3p.1 mediates acetylation of FOXO3a and enhances sorafenib-induced apoptosis by targeting SIRT1

**Fig. 6:**
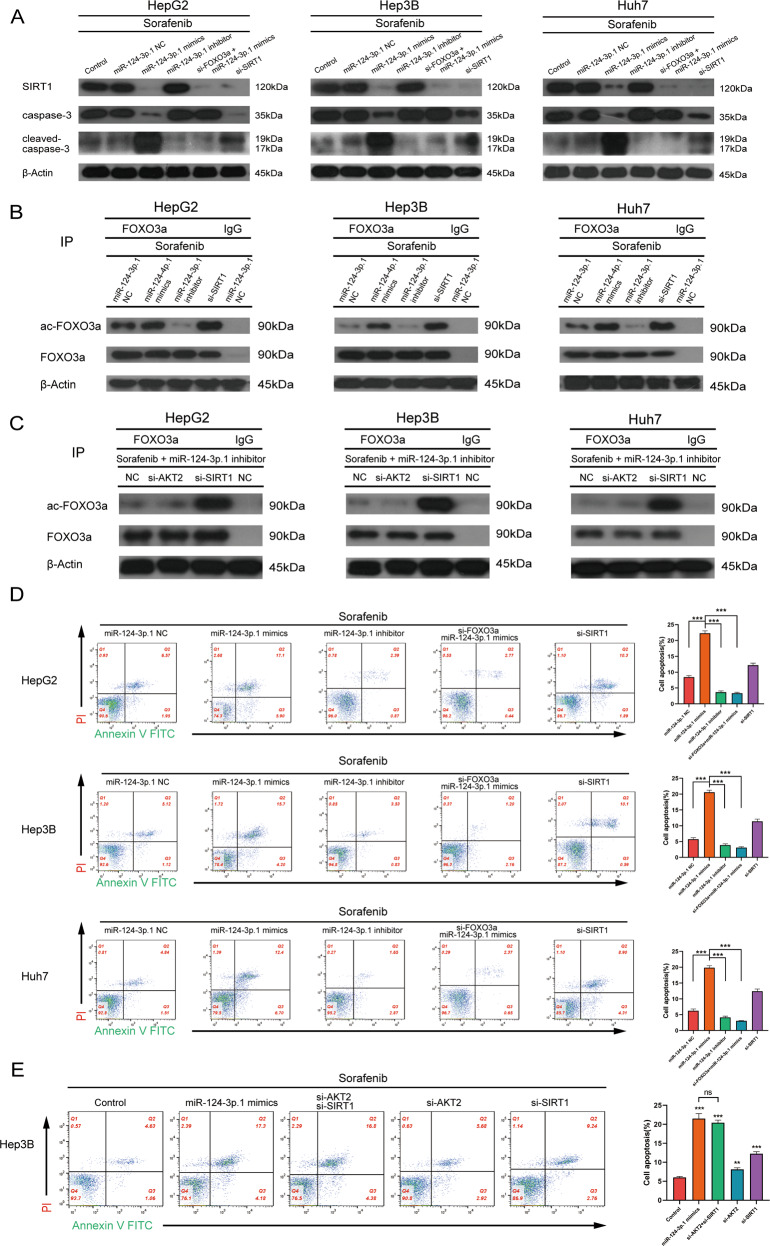
MiR-124-3p.1 mediates acetylation of FOXO3a and enhances sorafenib-induced apoptosis by targeting SIRT1. **A** Western blot analysis of SIRT1 (120 kDa), pro-caspase-3 (35 kDa) and cleaved-caspased-3 (19, 17 kDa) expression in HCC cells (cultured with sorafenib 10 µM, sorafenib 10 µM + miR-NC, sorafenib 10 µM + miR-124-3p.1 mimics, sorafenib 10 µM + miR-124-3p.1 inhibitor, sorafenib 10 µM + miR-124-3p.1 mimics + si-FOXO3a, or sorafenib 10 µM + si-SIRT1). **B** HCC cells were immunoprecipitated with FOXO3a antibody or IgG as control, and the level of acetyl-FOXO3a (90 kDa) and FOXO3a (90 kDa) were analyzed by Western blot (cultured with sorafenib 10 µM + miR-NC, sorafenib 10 µM + miR-124-3p.1 mimics, sorafenib 10 µM + miR-124-3p.1 inhibitor, or sorafenib 10 µM + si-SIRT1). **C** Western blot analysis of HCC cells cultured with sorafenib 10 µM + miR-124-3p.1 inhibitor + NC, sorafenib 10 µM + miR-124-3p.1 inhibitor + si-AKT2, or sorafenib 10 µM + miR-124-3p.1 inhibitor + si-SIRT1 and immunoprecipitated with FOXO3a antibody or IgG as a control. **D** Flow cytometric analysis of apoptosis rate in HCC cells cultured with sorafenib 10 µM+miR-NC, sorafenib 10 µM + miR-124-3p.1 mimics, sorafenib 10 µM + miR-124-3p.1 inhibitor, sorafenib 10 µM + miR-124-3p.1 mimics+si-FOXO3a, or sorafenib 10 µM + si-SIRT1 (*t*-test). **E** Flow cytometric analysis of apoptosis rate in Hep3B cells cultured with sorafenib 10 µM, sorafenib10 µM + miR-124-3p.1 mimics, sorafenib10 µM+si-AKT2 + si-SIRT1, sorafenib 10 µM + si-AKT2, or sorafenib 10 µM + si-SIRT1 (*t*-test). Data represent mean ± SD (*n* = ≥3 experiments). *P < 0.05, **P < 0.01, ***P < 0.001.

It has been confirmed previously that SIRT1 is negatively regulated by miR-124-3p.1. Moreover, it has been reported that SIRT1-mediated deacetylation of FOXO3a is related to apoptosis [31]. To investigate the potential involvement of SIRT1 in the reversal of sorafenib resistance mediated by miR-124-3p.1, we investigated the effects of sorafenib on three HCC cell lines following siRNA-mediated knockdown of SIRT1. Western blot analysis showed that the expression of ac-FOXO3a and cleaved-caspase-3 was significantly enhanced in all HCC cells following SIRT1 knockdown (Fig. 6A, B). Compared to the miR-NC group, SIRT1 was markedly downregulated in the miR-124-3p.1 mimics group, while the expression of ac-FOXO3a and cleaved-caspase-3 was upregulated, indicating an enhanced level of apoptosis (Fig. 6A, B).

To further investigate whether SIRT1 enhanced sorafenib-induced apoptosis by regulating FOXO3a, HCC cells were co-transfected with miR-124-3p.1 mimics and si-FOXO3a. Compared to the cells transfected with miR-124-3p.1 mimics alone, the expression of cleaved-caspase-3 in the combined treatment group was significantly decreased, and the pro-apoptotic effect of sorafenib was reversed (Fig. 6A). However, in the miR-124-3p. 1 inhibitor group, the inhibition of SIRT1 expression was alleviated, and the expression levels of ac-FOXO3a and cleaved-caspase-3 were also decreased (Fig. 6A, B). We also demonstrated that miR-124-3p.1 induced acetylation of FOXO3a by targeting SIRT1. After treatment of HCC cells with Sorafenib + miR-124-3p.1 inhibitor, the expression of ac-FOXO3a in si-SIRT1 group was significantly higher than that in si-AKT2 group (Fig. 6C). In addition, the results of flow cytometric analysis of cell apoptosis were consistent with the Western blot data. That is, HCC cell apoptosis was significantly higher in the miR-124-3p.1 mimics group, and this effect was reversed FOXO3a silencing (Fig. 6D). These data suggested that the expression of ac-FOXO3a regulated by SIRT1 is the key step in the mechanism by which miR-124-3p.1 promotes sorafenib-induced apoptosis.

We then compared the effects of transfection of miR-124-3p.1 mimics, si-AKT2/si-SIRT1 and si-AKT2 + si-SIRT1 on sorafenib-induced apoptosis in Hep3B cells. Knockdown of either AKT2 or SIRT1 promoted sorafenib-induced apoptosis, although compared to si-AKT2, si-SIRT1 had a more marked effect, suggesting that SIRT1 is the most important factor that regulates sorafenib-induced apoptosis (Fig. 6E). In addition, the cells transfected with miR-124-3p.1 mimics or si-AKT2 + si-SIRT1 showed much greater sensitivity to sorafenib than the cells transfected with si-SIRT1, although there was no significant difference between the two groups (Fig. 6E). This phenomenon indicated that miR-124-3p.1 significantly enhanced the apoptosis-promoting effect induced by SIRT1 by targeting AKT2 simultaneously, resulting in a synergistic effect.

### MiR-124-3p.1 enhances the curative efficacy of sorafenib in HCC in vivo

**Fig. 7:**
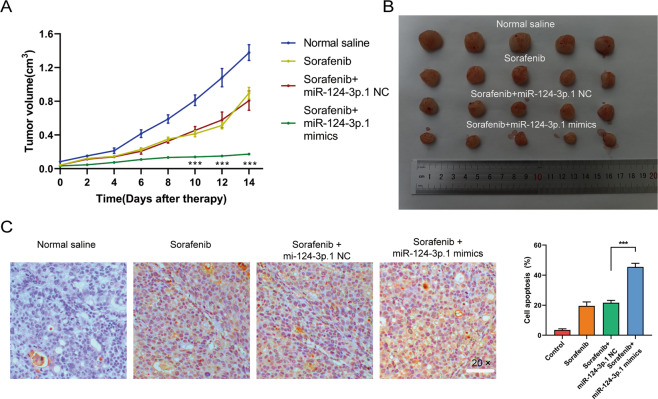
MiR-124-3p.1 enhances the curative efficacy of sorafenib in HCC in vivo. **A** Tumor regression rates were by tumor volume in nude mice (normal saline, sorafenib 10 µM, sorafenib 10 µM + miR-NC, sorafenib 10 µM + miR-124-3p.1 mimics; *t*-test). **B** Representative images of xenograft tumors in nude mice 2 weeks after different treatments. **C** TUNEL assay of cell apoptosis in the Hep3B tumors excised from nude mice after treatment (normal saline, sorafenib 10 µM, sorafenib 10 µM + miR-NC, sorafenib 10 µM + miR-124-3p.1 mimics; *t*-test). Data represent mean ± SD (*n* = ≥3 experiments). *P < 0.05, **P < 0.01, ***P < 0.001.

Next, we investigated the role of miR-124-3p.1 in the antitumor effects of sorafenib treatment in vivo using a nude mouse subcutaneous Hep3B xenograft model. As shown in Fig. 7A, the volume of subcutaneous tumors in the sorafenib treated group was significantly smaller than the those in the normal saline control group and the tumor size was further reduced in the miR-124-3p.1 mimics transfected group. The mice were euthanized after 2 weeks of treatment, and the subcutaneous xenografts were dissected and analyzed (Fig. 7B). TUNEL staining of apoptosis in Hep3B tumors indicated that sorafenib combined with miR-124-3p.1 mimics induced significantly more apoptosis than treatment with sorafenib alone (Fig. 7C).

## Discussion

The multikinase inhibitor sorafenib, which is one of the few systemic treatment options for advanced HCC, blocks tumor cell proliferation by inhibiting the activity of kinases in the Ras/Raf/MEK/ERK signaling pathway and inhibiting angiogenesis through targeting of c-Kit, VEGFR, PDGFR-β, and other tyrosine kinases, although its clinical efficacy is restricted by drug resistance [32, 33]. Recently, miR-124-3p.1 has been reported to function as a tumor suppressor in breast cancer by targeting CBL [34]. In addition, Xia et al. demonstrated that miR-124 inhibits cell proliferation in gastric cancer through downregulation of SPHK1 [35]. In our study, we revealed differences in the expression levels of miR-124-3p.1 between HCC tissues and normal liver tissues, as well as various HCC cell lines (Fig. 1A, B, Supplementary Fig. 1). Moreover, miR-124-3p.1 was related to early recurrence in HCC. Univariate and multivariate analyses of prognostic factors for recurrence-free survival revealed that miR-124-3p.1 represents a potential predictor of early HCC recurrence in patients undergoing curative surgery (Fig. 1C). Our research also showed that the differential expression of miR-124-3p.1 was related to the sorafenib sensitivity and miR-124-3p.1 overexpression promoted sorafenib-induced apoptosis (Fig. 2).

In this study, we demonstrated that miR-124-3p.1 binds directly to the 3′-UTRs of *AKT2* and *SIRT1* to inhibit their transcription (Fig. 3). In addition to acting as downstream targets of miR-124-3p.1, AKT2 and SIRT1 are also upstream regulators of FOXO3a, an important TF involved in the regulation of cellular stress responses. First, we verified the relationship between FOXO3a and sorafenib. FOXO3a is a member of the FOXO subfamily of forkhead TFs and plays an important role in apoptosis, proliferation, cell cycle progression, DNA damage and tumorigenesis [20]. It has been reported that FOXO3a is involved in the mechanisms underlying the anticancer effects of agents such as paclitaxel, cisplatin, imatinib [36–38]. We identified FOXO3a as a key factor in the anticancer effects of sorafenib treatment, and knockdown of FOXO3a significantly reduced sorafenib-induced apoptosis (Fig. 4C, D). In contrast, FOXO3a overexpression enhanced sorafenib-induced apoptosis (Fig. 4C, D). However, our previous studies have shown that FOXO3a enhanced sorafenib resistance by inducing autophagy of HCC cells in a hypoxic microenvironment [39]. Therefore, even in the same tumor, FOXO3a may play different roles in the regulation of stress. This may be attributed to different microenvironments, in which FOXO3a is induced to regulate different downstream target genes in nuclear transcription. Therefore, the selective epigenetic regulation of FOXO3a that induces the transcription of apoptosis-related genes to the greatest extent and increases cell apoptosis, is highlighted as a new model for alleviating sorafenib resistance. In addition to transcriptional regulation, FOXO3a is also regulated by post-translational modification (PTM), which largely determines the cell behavior regulated by FOXO3a. Thus, in subsequent studies, we focused on the PTMs of FOXO3a and explored their interaction with sorafenib-induced apoptosis.

**Fig. 8:**
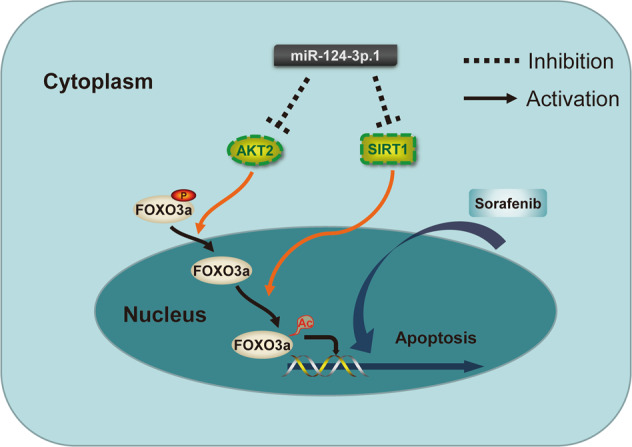
Schematic diagram. Schematic diagram of the predicted mechanisms implicated in the response of HCC cell to sorafenib and miR-124-3p.1.

Recent studies have indicated that AKT2 is associated with the prognosis of HCC and is responsible for the phosphorylation of FOXO3a [16]. In our study, we demonstrated that miR-124-3p.1 targets AKT2, thereby reducing the expression of p-FOXO3a and inducing FOXO3a nuclear localization (Fig. 5). Similar to phosphorylation, acetylation is another form of PTM that regulates FOXO3a in the nucleus. Wang et al. demonstrated that SIRT1 protects cells from apoptosis by increasing the ubiquitination of FOXO3a [40]. In addition, SIRT1 has been shown to decrease the expression of pro-apoptotic genes by deacetylating FOXO3a [31]. Compared to the corresponding adjacent noncancerous liver parenchyma, SIRT1 is frequently overexpressed in HCC biopsies and has been shown to stimulate oncogenesis and promote MDR in HCC [19, 41–44]. In addition, hypermethylated in cancer 1 and p53 negatively regulate SIRT1 mRNA transcription and are often mutated or dysfunctional in HCC [45]. In the current study, we have revealed the involvement of SIRT1 in the occurrence and development of HCC and the development of drug resistance. The decreased inhibition of SIRT1 transcription in HCC may account for its overexpression. We confirmed that miR-124-3p.1 expression was downregulated in HCC cells, and its expression was positively correlated with sorafenib-induced apoptosis (Figs. 1 and 2). Furthermore, as an upstream negative regulator of SIRT1, miR-124-3p.1 significantly decreased sorafenib-induced apoptosis by deacetylating FOXO3a (Fig. 6A, B). Further studies suggested that transfection with miR-124-3p.1 mimics promoted Hep3B cells apoptosis more significantly than knockout of AKT2 or SIRT1 alone (Fig. 6E). Based on these results, we concluded that miR-124-3p.1 induces translocation of FOXO3a to the nucleus by targeting AKT2. Subsequently, the increased intranuclear expression of FOXO3a further enhanced the expression of ac-FOXO3a, which was induced via miR-124-3p.1 by targeting SIRT1, ultimately enhancing tumor cell apoptosis. Overall, miR-124-3p.1 enhanced the sorafenib-induced apoptosis in HCC cells by regulating the phosphorylation and acetylation levels of FOXO3a, which play synergistic roles in promoting the apoptosis. A schematic diagram of the predicted mechanisms implicated in the response of HCC cell to sorafenib and miR-124-3p.1 is shown in Fig. 8.

In summary, we verified our hypothesis that FOXO3a is a key downstream factor in the mechanism by which sorafenib-induced apoptosis is regulated in HCC, and different PTMs of FOXO3a mediate different epigenetic regulation. We also revealed that miR-124-3p.1 is a potential predictor of early recurrence and sorafenib sensitivity in HCC patients undergoing curative surgery. MiR-124-3p.1 regulates the PTM of FOXO3a by simultaneously targeting AKT2 and SIRT1, a coordinate regulation mode that results in sensitization of HCC to sorafenib, thus, providing a new target to reduce resistance to sorafenib in HCC.

## Materials and methods

### Cell lines and patient specimens

Different types of the human HCC cell lines with high (HepG2) and low (Hep3B, Huh-7, LM-3, MHCC97L and MHCC97H) expression of miR-124-3p.1 were purchased from the Shanghai Institute for Biological Science (Shanghai, China). All cell lines were cultured in Dulbecco’s modified Eagle’s medium (DMEM, Gibco, Carlsbad, CA, USA) containing 10% fetal bovine serum (Gibco) and 1% penicillin/streptomycin at 37 °C under 5% CO_2_.

To investigate miR-124-3p.1 expression in HCC in vivo, we obtained tumor tissues and corresponding paracancerous tissues from 121 primary HCC patients (exclusion of extrahepatic metastasis, severe hepatic and renal insufficiency, and pregnant women) undergoing surgery at Lihuili Hospital affiliated to Ningbo University (China) between December 2016 and January 2018. All patients provided written informed consent prior to participation in this study and all experimental protocols were approved by the Ethics Committee of Lihuili Hospital affiliated to Ningbo University. The two-year disease-free survival was measured from the date of resection to the date of tumor recurrence.

### Drugs and antibodies

Sorafenib was purchased from Selleck (Houston, TX, USA). Primary antibodies for the detection of total-FOXO3a (t-FOXO3a, 12829S), p-FOXO3a (Ser253, 9466S), total-AKT2 (t-AKT2, 3063S), p-AKT2 (Ser473, 4060S), total-SIRT1 (2496S), acetylated-lysine (9441S), IgG (2985S), caspase-3 (14220S), and cleaved-caspase-3 (9661S) in Western blot, immunoprecipitation and immunofluorescence analyses were obtained from Cell Signaling (Danvers, MA, USA). The HRP-conjugated secondary antibodies were purchased from Beijing ZhongShan Biotechnology Company (Beijing, China). Anti-rabbit Alexa Fluor 555 (AF555) secondary antibody was purchased from Invitrogen (Carlsbad, CA, USA).

### Detection of miR-124-3p.1 expression

Relative expression of miR-124-3p.1 was determined by quantitative real-time polymerase chain reaction (qRT-PCR) analysis. Total RNA (including miRNAs) was extracted from cells using TRIzol reagent (Invitrogen) according to the manufacturer’s protocol. For analysis of miR-124-3p.1 expression, reverse transcription was performed to generated cDNA and amplified using the Mir-X miRNA First Strand Synthesis kit (TaKaRa, CA, USA). U6 snRNA was probed as a loading control. Measurement was carried out by Roche LightCycler 480 (Basel, Switzerland). The relative fold-change in mRNA expression was calculated according to the 2^−ΔΔCt^ method.

### Transfection

Mature human miR-124-3p.1 mimics (5′-UAAGGCACGCGGUGAAUGCC-3′), negative control oligonucleotides (NCO, 5′-UUCUCCGAACGUGUCACGUTT-3′) and the miR-124-3p.1 inhibitor (5′-UUGGCAUUCACCGCGUGCCUUA-3′) were purchased from GenePharma Co. Ltd (Shanghai, China). HCC cells 2 × 10^7^ were plated into six-well plates and transfected with a scrambled negative control siRNA (NC-siRNA), FOXO3a-siRNA (FOXO3a-homo-1886, 5′-CCAGGGAAGUUUGGUCAAUTT-3′; FOXO3a-homo-1620, 5′-GGACCUUCAUCUCUGAACUTT-3′; FOXO3A-homo-2370, 5′-GACCCUCAAACUGACACAATT-3′), AKT2-siRNA (AKT2-homo-395, 5′-GCUCCUUCAUUGGGUACAATT-3′; AKT2-homo-848, 5′-GCGGAAGGAAGUCAUCAUUTT-3′; AKT2-homo-1519, 5′-GGUUCUUCCUCAGCAUCAATT-3′), SIRT1-siRNA (SIRT1-homo-606, 5′-CGGGAAUCCAAAGGAUAAUTT-3′; SIRT1-homo-512, 5′-CCAUCUCUCUGUCACAAAUTT-3′; SIRT1-homo-1216, 5′-CCAAGCAGCUAAGAGUAAUTT-3′) (all purchased from GenePharma, Shanghai, China), FOXO3a-TM (FOXO3a overexpression plasmid, purchased from Addgene, Cambridge, MA, USA) using Lipofectamine 2000 (Invitrogen) according to the manufacturer’s instructions. After 6 h, the medium (Opti-MEM, Gibco) was replaced with complete culture medium. The transfected HCC cells were incubated in different groups for the indicated time periods. To avoid off-target effects, we confirmed the efficacy of the interfering RNA, and selected the siRNA with the highest interference efficiency for use in subsequent experiments (Supplementary Fig. 3). All experiments were performed in 48 h after transfection.

### Cell viability assay

Cell viability was measured using Cell Counting Kit-8 (CCK-8, Dojindo, Kumamoto, Japan) according to the manufacturer’s instructions. HCC cells (8 × 10^3^) were plated into 96-well plates with conditioned media containing different concentrations of sorafenib for the indicated time periods. After incubation with CCK-8 solution for 3 h, the absorbance of the wells was measured at 450 nm using an MRX II microplate reader (Dynex, Chantilly, VA, USA). Relative cell viability was calculated as a percentage of untreated control HCC cells. Cell proliferation assays were performed using the Click-iT 5-ethynyl-20-deoxyuridine (EdU) Imaging Kit (Invitrogen) according to the manufacturer’s instructions.

### Immunoprecipitation and western blot analysis

Cells were lysed in NP-40 lysis buffer. Protein concentrations were measured using the BCA protein assay reagent (Thermo Fisher Scientific Inc., Rockford, IL, USA). The cell lysates (500 µg protein) were immunoprecipitated with FOXO3a antibody overnight followed by incubation with a 50% slurry of protein G sepharose beads for 3 h at 4 °C. The beads were washed three times with the lysis buffer, and the immunoprecipitated protein complexes were resuspended in 2× Laemmli sample buffer for Western blot analysis. The prepared protein lysates were then denatured by boiling and separated by 10% sodium dodecyl sulfate-polyacrylamide gel electrophoresis and transferred to polyvinylidene fluoride membranes (Millipore, Billerica, MA, USA). The membranes were then incubated with relevant primary antibodies overnight at 4 °C. Subsequently, the membranes were incubated with the appropriate HRP-conjugated secondary antibody for 2 h. Protein bands were developed by chemiluminescence (GE Healthcare, Piscataway, NJ, USA) and visualized using an autoradiography kit (Kodak, Rochester, NY, USA).

### Dual-luciferase reporter assay

The 3′-UTR segments of AKT2 and SITR1 including the WT or the mutant miR-124-3p binding sites were cloned downstream of the luciferase reporter in the pmirGLO Dual-Luciferase miRNA Target Expression Vector (Promega, Madison, USA), between the *Sac*I and *Sal*I sites and verified by sequencing. HEK 293T cells (10^5^) were plated into 24-well plates and transfected with 50 nM miR-124-3p.1 or NC and 100 ng of the luciferase vector (pmirGLO). Cells were harvested 48 h after transfection and the relative luciferase activity was measured using the Dual-Glo luciferase assay kit (Promega).

### RNA-binding protein immunoprecipitation assay

RIP was completed with an EZ-Magna RIP kit (Millipore, Billerica, MA, USA). Hep3B cells were harvested and lysed with lysis buffer. The cell extracts were incubated with magnetic beads coated with anti-AKT2/SIRT1 antibodies or control. Subsequently, total RNA was extracted from the magnetic beads. The co-precipitated RNAs were detected by RT-qPCR.

### Detection of apoptosis

Cell apoptosis was measured by flow cytometric analysis of annexin V/PI double staining. Briefly, treated liver cancer cells were collected after culture overnight in DMEM medium, and resuspended in 100 μm annexin V binding buffer (Dojindo, Kumamoto, Japan) supplemented with 1% annexin V-FITC and PI. After incubation at room temperature for 15 min in the dark, the cells were analyzed by flow cytometry (BD FACSCanto II, NJ, USA).

### Immunofluorescence assay

HCC cells cultured a glass cell culture dish were fixed with 4% paraformaldehyde and then permeabilized in 0.5% Triton X-100. After blocking with 5% bovine serum albumin, the HCC cells were incubated with anti-FOXO3a primary antibody overnight at 4 °C and then incubated with anti-rabbit AF555 secondary antibody. DAPI was used for nuclear counterstaining. Immunofluorescent images were captured using a confocal laser scanning microscope (Olympus, Tokyo, Japan).

### In vivo experiments

Animal studies were conducted in compliance with the Guide for the Care and Use of the Animal Ethics Committee of Ningbo University. Male nude mice (aged 3–4 weeks) were purchased from Shanghai Experiment Animal Center (Shanghai, China). Prepared Hep3B cells (1 × 10^6^) were injected subcutaneously into the right axillary fossa of each mouse. Tumor length (L) and width (W) were measured by sliding caliper every 2 days, and tumor volumes were calculated according to the formula (L × W^2^)/2 [46]. Sorafenib (60 mg/kg) therapy was initiated when tumor volumes reached 50–100 mm^3^ and delivered intragastrically every 2 days for 2 weeks. At least five nude mice were randomly allocated to each group. The miR-124-3p.1 mimics was injected into the tumor once a week. After 2 weeks of treatment, mice were euthanized by cervical dislocation. The tumors were dissected from each mouse and prepared for subsequent experiments.

### TUNEL assay of xenograft tumor tissue

Tumor tissues from xenografts were excised and immediately fixed in formalin. TUNEL staining was performed using an In Situ Cell Death Detection Kit, POD (Roche, Germany) according to the manufacturer’s instructions. Images were acquired with a Nikon ECLIPSE E800 fluorescence microscope (Tokyo, Japan). The percentage of apoptotic cells was assessed in randomly selected fields at ×20 magnification.

### Statistical analysis

All the data were obtained from experiments with adequate sample sizes and presented as the mean ± standard deviation (SD). Statistical analysis was performed using Prism 8 (GraphPad, San Diego, CA, USA) and SPSS software version 20.0 (SPSS Inc., Chicago, IL, USA). Two-way ANOVA, one-way ANOVA, *χ*^2^ tests and the two-tailed unpaired Student’s *t* tests were used to assess the significance of the differences between groups. The Kaplan–Meier method and log-rank tests of survival were used to study the role of miR-124-3p.1 in HCC progression. *P* < 0.05 was set as the threshold for statistical significance. Each treatment was analyzed in at least three experiments.

## Supplementary information

Supplementary Table 1,Correlation between miR-124-3p.1 expression and HCC clinicopathological parameters.

Supplementary Table 2. Univariate analyses of prognostic factors for recurrence-free survival.

Supplementary Table 3. Multivariate analyses of prognostic factors for recurrence-free survival.

Supplementary Figure legends.

Supplementary Fig. 1. MiR-124-3p.1 expression in HCC cell lines was differently.

Supplementary Fig. 2. MiR-124-3p.1 alone made no effect on HCC cell viability.

Supplementary Fig. 3. Efficacy of si-RNA.

## Data Availability

The data that support the findings of this study are available from the corresponding author upon reasonable request.
