# Supplementary material for: MiRNA-124-3p.1 sensitizes hepatocellular carcinoma cells to sorafenib by regulating FOXO3a by targeting AKT2 and SIRT1
Source: Cell Death Dis. 2022 Jan 10;13(1):35. doi: 10.1038/s41419-021-04491-0 (PMC8748751; doi:10.1038/s41419-021-04491-0)
Supplement: Supplementary file 4 — Supplementary Figure legends. [file 41419_2021_4491_MOESM4_ESM.docx]

**Supplementary Fig. 1.** **MiR-124-3p.1 expression in HCC cell lines was differently.** qRT-PCR analysis of miR-124-3p.1 expression in HCC cells (HepG2, Hep3B, Huh-7, LM-3, MHCC97L and MHCC97H; one-way ANOVA). Data represent mean ± SD (n = ≥3 experiments). **P* < 0.05, ***P* < 0.01, ****P* < 0.001.

**Supplementary Fig. 2.** **MiR-124-3p.1 alone made no effect on HCC cell viability.** Relative viability of HCC cells cultured with miR-NC, miR-124-3p.1 mimics, sorafenib 10 µM+miR-NC, or sorafenib 10 µM+miR-124-3p.1 mimics (two-way ANOVA). Data represent mean ± SD (n = ≥3 experiments). **P* < 0.05, ***P* < 0.01, ****P* < 0.001.

**Supplementary Fig. 3.** **Efficacy of si-RNA.** (A) qRT-PCR analysis of FOXO3a mRNA in Hep3B cells transfected with si-FOXO3a (control, FOXO3a-homo-1886, FOXO3a-homo-1620, FOXO3A-homo-2370; *t*-test). (B) qRT-PCR analysis of AKT2 mRNA in Hep3B cells transfected with si-AKT2 (control, AKT2-homo-395, AKT2-homo-848, AKT2-homo-1519; *t*-test). (C) qRT-PCR analysis of SIRT1 mRNA in Hep3B cells transfected with si-SIRT1 (control, SIRT1-homo-606, SIRT1-homo-512, SIRT1-homo-1216; *t*-test). Data represent mean ± SD (n = ≥3 experiments). **P* < 0.05, ***P* < 0.01, ****P* < 0.001.
