# Supplementary figures and images for: MiRNA-124-3p.1 sensitizes hepatocellular carcinoma cells to sorafenib by regulating FOXO3a by targeting AKT2 and SIRT1
Source: Cell Death Dis. 2022 Jan 10;13(1):35. doi: 10.1038/s41419-021-04491-0 (PMC8748751; doi:10.1038/s41419-021-04491-0)

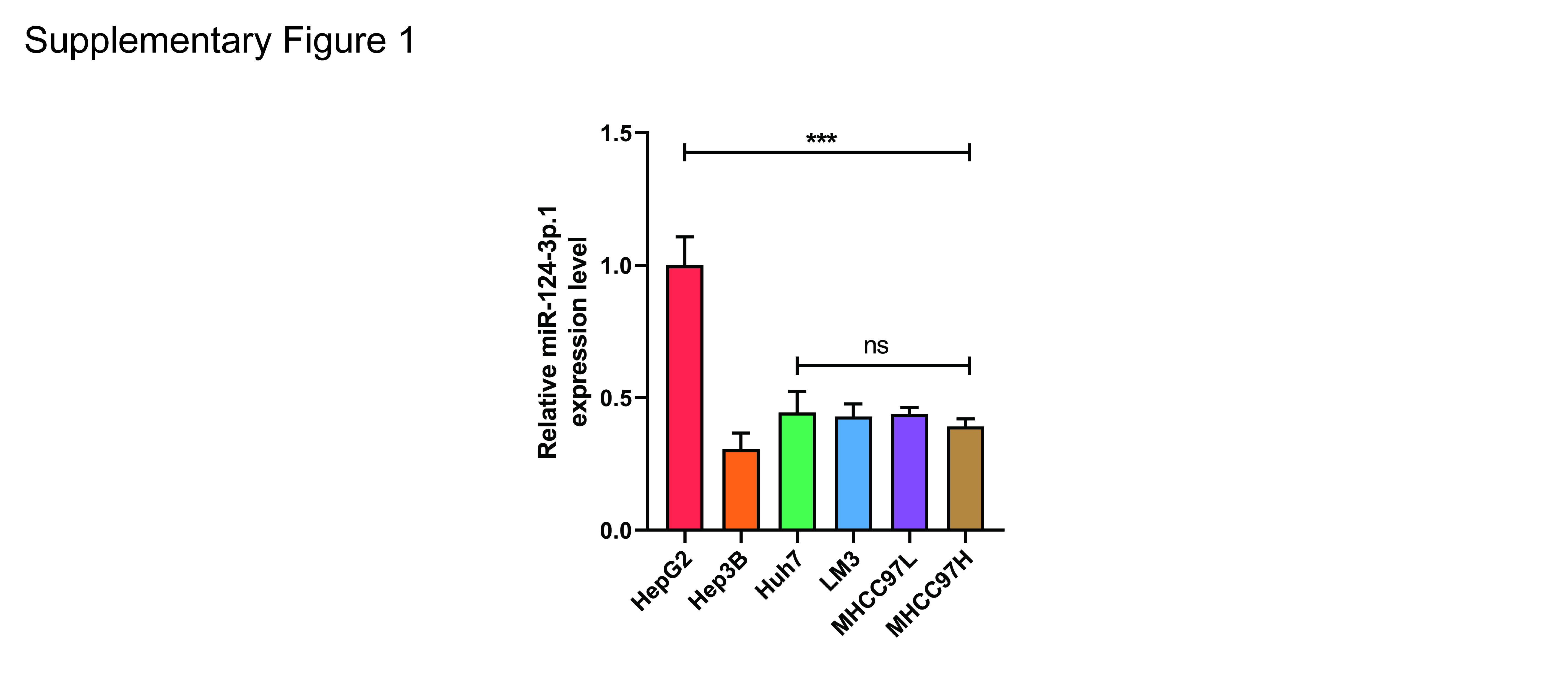

Supplement: Supplementary file 5 — Supplementary Fig. 1. MiR-124-3p.1 expression in HCC cell lines was differently. [file 41419_2021_4491_MOESM5_ESM.tif]

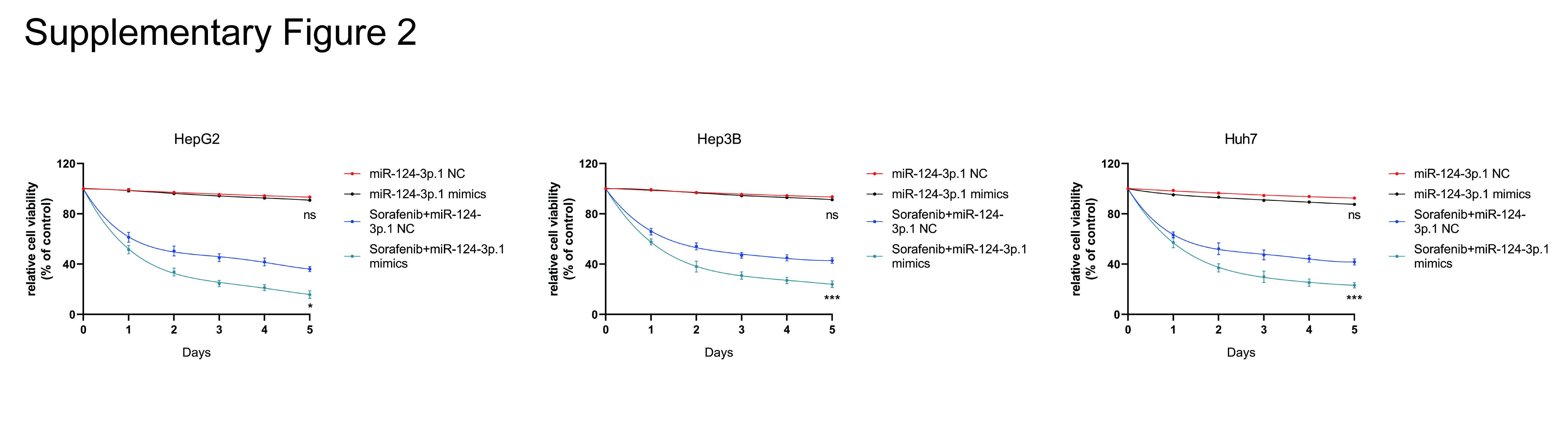

Supplement: Supplementary file 6 — Supplementary Fig. 2. MiR-124-3p.1 alone made no effect on HCC cell viability. [file 41419_2021_4491_MOESM6_ESM.tif]

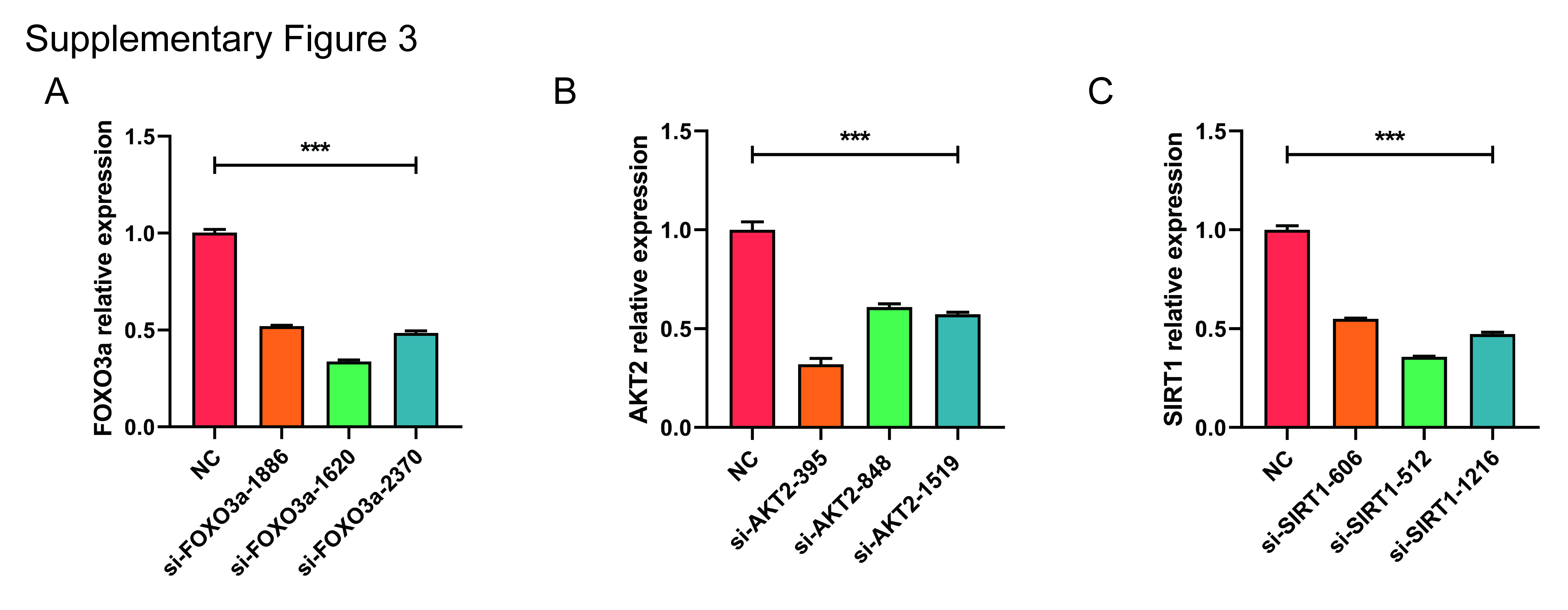

Supplement: Supplementary file 7 — Supplementary Fig. 3. Efficacy of si-RNA. [file 41419_2021_4491_MOESM7_ESM.tif]
